# Geometry Dependent Evolution of the Resonant Mode in ZnO Elongated Hexagonal Microcavity

**DOI:** 10.1038/srep19273

**Published:** 2016-01-14

**Authors:** Hongxing Dong, Yang Liu, Shulin Sun, Jingzhou Li, Jinxin Zhan, Zhanghai Chen, Long Zhang

**Affiliations:** 1 Key Laboratory of Materials for High-Power Laser, Shanghai Institute of Optics and Fine Mechanics, Chinese Academy of Science, Shanghai, 201800, China; 2 Department of Optical Science and Engineering and Key Laboratory of Micro and Nano Photonic Structures (Ministry of Education), Fudan University, Shanghai 200433, China; 3 State Key Laboratory of Surface Physics and Department of Physics, Fudan University, Shanghai, 200083, China

## Abstract

We have developed a novel but simple approach to obtain ZnO microcombs with parallelogram stems and elongated hexagonal branches. We found that the present elongated hexagonal microcavity exhibited quite different features for its optical resonant modes due to the broken hexagonal symmetry. The resonant mode evolution of such microcavity was investigated systemically by using a spatially resolved spectroscopic technique. Theoretical analyses based on the plane wave mode and FEM simulations agreed well with the experimental results. We believe that our research allows us to have a deeper understanding of the controllable growth of novel optical cavities and the shape-dependent optical resonant modes.

One-dimensional hexagonal microwire/rod whispering gallery mode (WGM) microcavities, as one of the most important type of optical cavities, have attracted great attention for their excellent optical properties and wide applications^1^,^2^,^3^. In a hexagonal WGM resonator, light wave can be mostly confined inside the microcavity due to multiple total internal reflections at the six boundaries of the cavity. Such efficient low-dimensional optical limitations are very important for both physical optics and miniature optoelectronic devices. Up to now, many microwires/rods such as ZnO^4^,^5^,^6^, ZnS^7^, GaN^8^, etc., which can be used as hexagonal WGM microcavity, have been developed. It has been demonstrated that the cross sectional geometry of the microcavity is one of the most critical factors for efficient optical resonant modes. Investigation and clarify the effects of the cross sectional geometry on optical resonant modes in microcavity are therefore important and necessary. Although some irregular hexagonal microcavity can be obtained, the effects has not been systematically studied and clarified^9^,^10^. And, in fact, almost all the studies are focused on the optical microcavities with regular hexagonal cross section, which lead to the research in this field is very limited. How to design and synthesis of the microcavity with gradual and imperfect asymmetrical hexagonal cross section, and further investigate the dependence of optical resonant modes on microcavity cross sectional shape has become one of important and interesting issue.

Zinc oxide (ZnO), one of the most promising candidate materials for hexagonal WGM optical resonators, has drawn much attention due to its wide band gap (3.37 eV) and large exciton binding energy (~60 meV). Many ZnO microstructures with different morphologies, such as microwires^11^, microdisks^12^, microcombs^13^, microtubes^14^, etc., have been fabricated. Most studies on WGM optical resonators are based on the ZnO microwires with regular hexagonal cross section^15^,^16^,^17^. In fact, hierarchical ZnO microstructure such as microcombs is an important building block for nanoscale devices owing to its exceptional structural characteristics, which has found applications in micro-gratings, photodetector arrays, etc.^13^,^18^. Compared with the growth of one-dimensional ZnO hexagonal nanocrystals, the complex and diverse crystal growth process of such hierarchical nanostructures is more helpful to break the traditional growth model. This is why most of the synthesized ZnO micro/nanocombs have a variety of stems and branches with different morphologies^19^,^20^. It may be open up the possibility of studying experimentally the effects of microcavity geometry on hexagonal WGM optical resonant modes. On the other hand, as we know, to be used as microcavity, micro/nanostructure should meet the requirements of high crystal quality, regular geometry, smooth surfaces and size approximate to wavelength. However, to the best of our knowledge, practical examples of micro/nanocombs in the form of irregular hexagonal WGM microcavities are very rare.

In the present study, we reported the synthesis of the novel single-crystalline ZnO microcombs with parallelogram stems and elongated hexagonal branches, which can effectively control the light field in two dimensions. Growth mechanism of these ZnO microcombs was investigated systematically, and the ratio of Sb_2_O_3_ in the source material was confirmed to be the key factor in the growth process. Compared with the conventional hexagonal WGM microcavity, the present microcavity with elongated hexagonal cross section exhibited quite different features. The effects of the cross sectional geometry on optical resonant modes in microcavity were mapped directly by using the spatially resolved spectroscopic technique. And, the experimental results further confirmed by Finite Element Method (FEM) simulations. Such ZnO microcomb microcavity offers a promising test-bed for investigating new optical physics and developing novel micro-optoelectronic devices.

## Experiments and measurements

Single-crystalline ZnO microcombs with parallelogram stems and elongated hexagonal branches were synthesized in a conventional horizontal tube furnace. In our experiment, a mixture of commercial ZnO (99.99%), Sb_2_O_3_ (99.99%) and graphite (99.99%) powders with a ratio of 0.1 g : 0.005 g : 0.1 g was put into a small quartz boat. Si wafer with 20 nm Au film was used as substrate and covered on the source material. The quartz boat was then placed in the center of the tube furnace and the temperature was raised to 1000 °C to reaction for 50 mins with a flow of mixed gas of N_2_ (80 sccm) and O_2_ (5 sccm). After the furnace was cooled to room temperature, a large quantity of crystal-like products was obtained on the substrate. The morphologies of the products were characterized by field emission scanning electron microscopy (FE-SEM, Zeiss Auriga S40), and etching process was carried out by the same SEM with Ga ions focused ion beam (FIB) accessory. The composition and structure of the microcombs were measured by energy dispersive X-ray spectroscopy (EDS) and X-ray diffraction (XRD, PANalytical Empyrean with Cu Ka radiation (λ = 1.5418 Å)). The thickness of the ZnO microcombs was measured through optical profilometer (Bruker, Wyko NT9100). The optical properties of the ZnO microcombs were carried out using a confocal microphotoluminescence system (JY LabRAM HR800 UV). He-Cd laser of 325 nm was used as the excitation source. FEM simulations were performed using an commercial software (COMSOL Multiphysics).

## Results and Discussions

**Figure 1:**
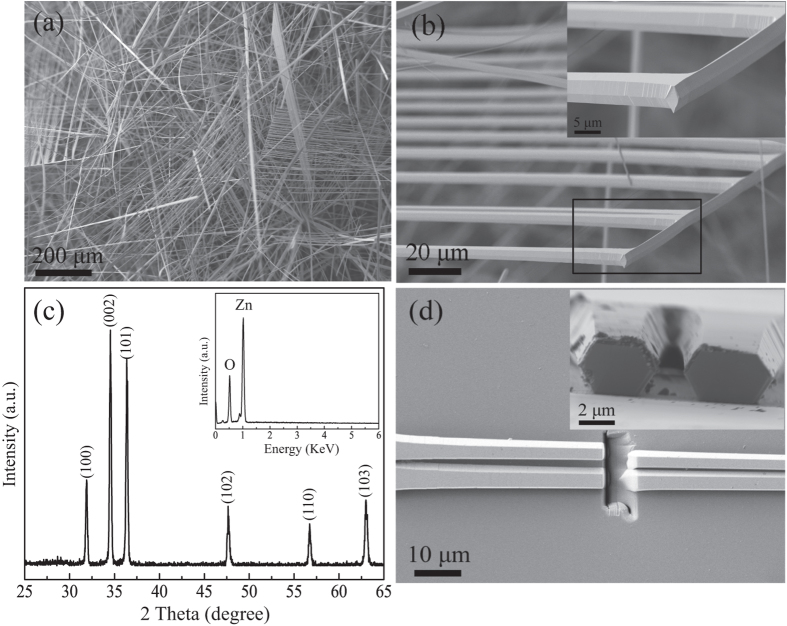
Microstructure of the samples. (**a**) The general morphology of the ZnO microcombs. (**b**) The typical SEM of one single ZnO microcomb and enlarged SEM of the top of the microcomb stem from rectangular region. (**c**) XRD pattern and EDS spectrum (inset) of the obtained ZnO microcombs. (**d**) SEM images of a single microcomb dispersed and etched by FIB on Si wafer. Inset is the cross section view of the etched branches.

Figure 1a displays the SEM image of the sample obtained on the substrate, clearly showing some comb-like microstructures. The detailed structure of the microcomb could also be resolved from high-magnification SEM image (Fig. 1b). It can be seen that the microcomb is composed of nanowire with parallelogram cross section stem and microwires with hexagonal cross section branches. A row of hexagonal microwires grow along one side of a parallelogram microwire stem. The diameter and length of both stem and branches were 2–6 μm and several hundreds of micrometers, respectively. The corresponding XRD pattern (Fig. 1c) shows that these novel ZnO microcombs are indexed to hexagonal wurtzite ZnO (JCPDS No. 36–1451). EDS spectrum of ZnO microcomb indicates that the sample is stoichiometric composition. To confirm the cross-section of these branches, FIB etching process was performed by SEM to cut off some branches, and the result was shown in Fig. 1d. It is interesting to find that the microwire branch has an elongated hexagonal cross-section with gradually decreased size along the c-axis, which is rarely reported in previous literatures. Such comb-like microstructures may provide an ideal system to further study optical resonant modes of hexagonal WGM microcavity.

**Figure 2:**
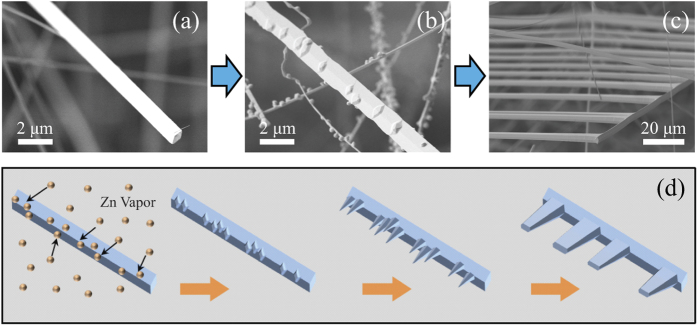
Time-dependent evolution of the sample morphology at different growth stages: (**a**) 20 min, (**b**) 35 min, (**c**) 50 min. (**d**) Schematic diagram of the growth process of the ZnO microcomb with parallelogram stem and elongated hexagonal branches.

To further understand the formation of such ZnO microcombs, their growth morphologies were studied in reaction time sequence. Figure 2a shows one typical ZnO microwire with parallelogram cross section which was obtained after reaction for 20 min. The microwire has smooth surface and regular parallelogram cross section with an angle of 45°, which has been illuminated as an effective wave-guided optical microcavity in our latest work^5^. As the reaction proceeded to 35 min (Fig. 2b), some small grains emerged on the surfaces of the microwires, which are the new nucleation sites of the branching microrods. Further increase of the reaction time to 50 min, ZnO microcombs are obtained, as shown in Fig. 2c. The Sb_2_O_3_ powder is crucial for the formation of initial ZnO microwires with parallelogram cross section, which has been illustrated in our study of 1D ZnO wave-guided optical resonators^5^. From the results one can deduced that the influence on the morphology of ZnO microstructure by Sb vapor gradually receded when the Sb_2_O_3_ powders were react completely at high temperature. Small grains began to grow on the surfaces of ZnO microwires, which will directly induce the growth of the hexagonal shaped branches. Generally, ZnO nano/microwire has hexagonal feature due to its intrinsic growth habit along the [0001] direction. For ZnO microcombs, the branches grow along [0001] direction, while the microwire stem grow in [2–1–10] direction. Such growth mode changes should be related to the introduction of the Sb element in the reaction process^21^. Based on the obtained results, the growth process of the ZnO microcombs is schematically illustrated in Fig. 2d. In the first stage of the reaction (0~20 min), Sb and Zn vapor were generated by the reaction of source material, which lead to the growth of microwire with parallelogram cross section. As the growth stage proceeded to about 35 min, the reaction of Sb_2_O_3_ and graphite will complete, while Zn vapor still transporting to the substrate. In the meantime, Zn vapor will condense on the surfaces of the microwires and form new nucleuses. The nucleuses will grow to hexagonal microwires without the influence of Sb. Moreover, a high temperature may accelerate the lateral growth of the ZnO nanowires, which lead to the neighboring ZnO nanowires at the same grain region easily combined with each other and grow as one microwire^22^. Thus, ZnO microcombs with elongated hexagonal, various diameter and random spacing branches were synthesized successfully.

**Figure 3:**
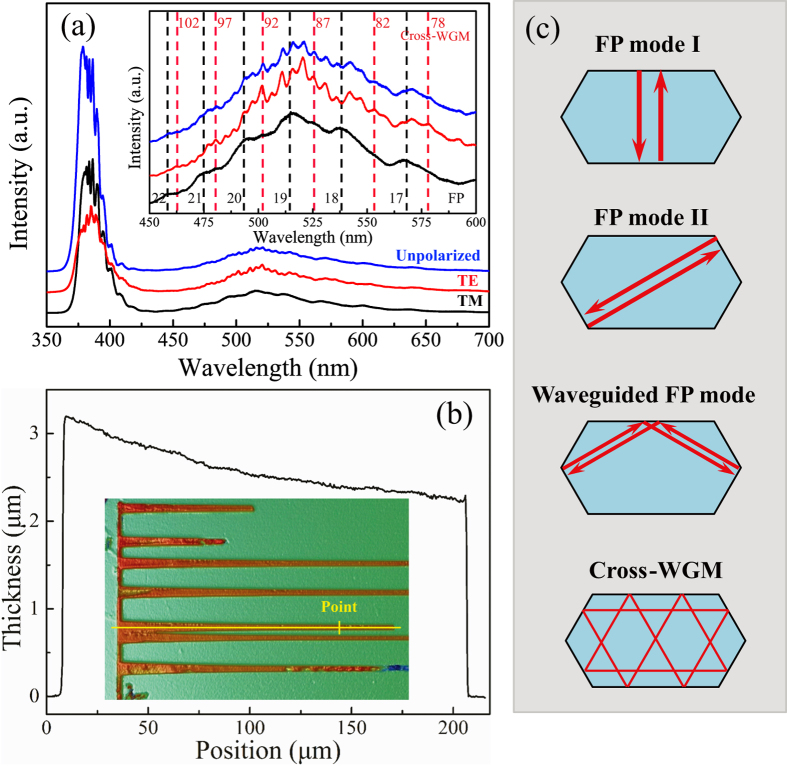
PL spectra with unpolarized, TE and TM polarized of individual branch of the selected ZnO microcomb. The inset is an enlarged view of the resonance modes between 450 to 600 nm. The two series of integers are the interference orders of the relevant resonant modes. (**b**) Thickness curve of the branch used in PL measurement (yellow line) and the spectra was detected at the point marked in the inset. (**c**) Three different resonant mode types can be formed in the elongated hexagonal cross section: simple FP modes (I, II), wave-guided FP modes and Cross-WGM.

Optical properties of individual ZnO microcombs were carried out with a microconfocal photoluminescence (PL) spectroscopic system. As shown in Fig. 3a, distinct resonant peaks can be observed in unpolarized (blue curve), TE-polarized (the electrical component of light E⊥c-axis, red curve) and TM polarized (E‖c-axis, black curve) PL spectra were taken from the point marked in the inset of Fig. 3b. These peaks correspond to the resonance of optical modes. It is noteworthy that the shape and periodic structure of the unpolarized and TE polarized resonant modes are different with the TM resonant modes, which can be clearly found from the enlarged view of the PL spectra (inset in Fig. 3a). The interesting phenomenon indicates that the resonant peaks belong to two different resonant modes at the same excitation position. It is known to us that the resonant modes depend on the geometrical structure and the effective length of the optical microcavity. To get precise size of the sample, the size and thickness of the microcomb were determined by SEM and optical profilometer, respectively. Figure 3b shows the detailed morphology of one single ZnO microcomb and the thickness curve of one microwire branch. The two different side lengths, R_1_ and R_2_, at the excitation spot position were determined as 1.40 μm and 3.32 μm, respectively. In the point of view of geometrical optics, three kinds of resonant cavity modes can be formed in this elongated hexagonal microcavity (Fig. 3c): (i) simple Fabry-Pérot (FP) modes (I and II) formed between the two opposite facets, (ii) wave-guided FP modes, formed between two unparallel short surfaces with an total internal reflection (TIR) at the interface of long edge, and (iii) cross whispering gallery mode (Cross-WGM), formed by crossing light beams with eight TIRs at the six interfaces. In order to determine the exact cavity modes related to the PL signals, adjacent peaks (520.2 nm and 525.3 nm from TE, 515.8 nm and 537.2 nm from TM) were selected to calculate the effective optical path length L by the following function:


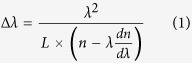


Here n is the refractive index of the ZnO. The factor 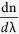 is the dispersion relation. The mode spacing Δλ between the two adjacent peaks is 5.1 nm and 21.4 nm, respectively. According to the refractive dispersion in our previous work^23^, the refractive indices (n_TE_ = 2.021 at 520.2 nm, n_TM_ = 2.020 at 515.5 nm) and the related λ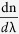 (−0.542 at 520.2 nm, −0.577 at 515.5 nm) were obtained. The calculated path lengths for TE and TM signals are about L_TE_ = 20.70 μm and L_TM_ = 4.79 μm. Based on geometrical optics and typical plane wave model, the effective length of these three kinds of resonant modes can be deduced to (i) 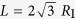 = 4.85 μm (FP mode I), 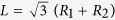 = 5.75 μm (FP mode II); (ii) 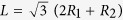 = 10.60 μm (Wave-guided FP); (iii) L = 9 R_1_ + 3 R_2_ = 22.56 μm (Cross-WGM). Obviously, the calculated effective length of FP mode I (4.85 μm) is exactly consistent with the calculated effective path lengths of TM signal (4.79 μm) according to equation (1). It can be concluded that the measured resonant modes from TM signal and the UV region of TE signal are attributed to the effect of the FP mode I type microcavity. We also noticed that the calculated effective path lengths of TE signal is not fit well with the Cross-WGM, which may be caused by the differences between the actual refractive indices of the ZnO microcombs and the cited refractive indices in the calculation process. Further calculations need to be done to identify the resonant model.

Firstly, the actual TM refractive index can be deduced from the FP model:


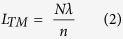


Where n is the refractive index of ZnO microcomb, N is the interference order of resonant mode number. The accurate wavelength-dependent refractive indices (n_TM_) were obtained from the best fit of the FP equation by varying N discretely and keeping the cavity length L within the experimental error. The accurate TM refractive indices can be fitted well with Sellmeier dispersion formula as follows:


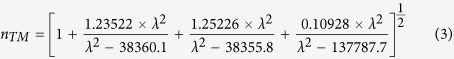


It is well know that the refractive indices for TE polarization are close to each other at the same wavelength in the visible region. So, the effective optical path length L of the resonant peaks from TE signal was further optimized by the accurate TM refractive indices. The mode spacing Δλ is 5.1 nm. The refractive index at the wavelength of 520.2 nm is about 2.030, and the relation λ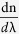 is about −0.347. We have obtained the effective length L of about 22.30 μm, which is consistent with the Cross-WGM mode (L = 22.56 μm). The results demonstrated that another resonant mode is attributed to the effect of the Cross-WGM type microcavity. Such complicated Cross-WGM model should be related to the elongated shape of the microwire structure. It is interesting to note that such optical resonant model is very different from previous theoretical analysis and experimental results^9^,^10^. The reason may be due to the size and extent of the microcavity deformation. Further discussion will be done in the following analyses.

Taking into account the number of TIRs and the resonant energy, the effective length can be calculated from the following equations:


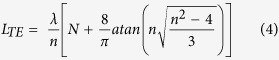


The interference orders of TE resonant modes can be obtained as N_TE_ = 76–105 in the range of 450–600 nm, and the result was shown in the inset of Fig. 3a as the two series of integers marked with dashed lines in the enlarged PL spectra. The accurate wavelength dependent TE refractive dispersions can also be calculated through the same process of n_TM_, the result fitted with Sellmeier dispersion function as follows:


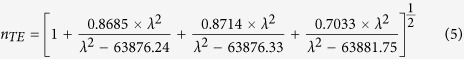


**Figure 4:**
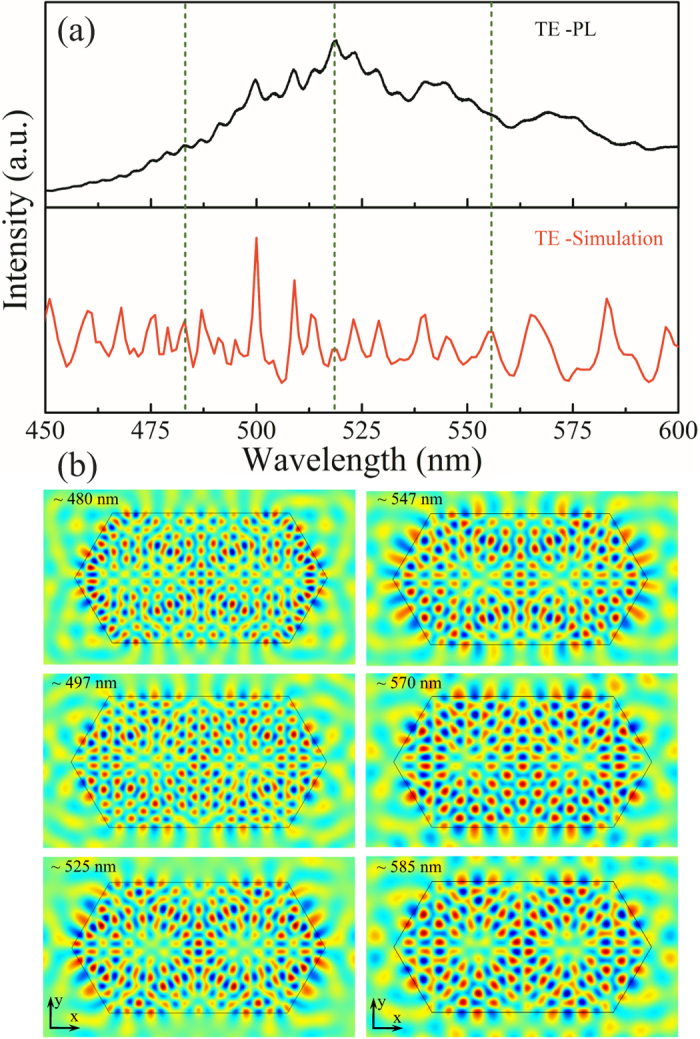
Electric field distribution for the resonant modes. (**a**) TE polarized PL spectra and the FEM simulated backward reflectance spectra of ZnO microcomb branch. (**b**) Simulated Hz field distributions of some resonance modes inside the elongated hexagonal microcavity at several resonance wavelengths (see insets) for TE polarization.

We then employed FEM simulations to identify the optical resonant modes in the ZnO cavity. A 2D model with the same edge length (R_1_ = 1.40 μm, R_2_ = 3.32 μm) as the elongated hexagonal cross section of fabricated sample was created. And its refractive index was chosen from the values described in equation (5). In our simulations, we shined a plane wave on the top surface of the microcavity and then detected its backward reflectance spectra. Perfectly matched layers were used for the surrounding boundaries to absorb the scattered electromagnetic fields of the cavity. As shown in Fig. 4a, the computed resonance peaks for TE polarization perfectly match with the experimentally measured results. We then performed eigenmode analysis simulations to further illustrate the mode field patterns of ZnO cavity at the resonance wavelengths. Here, the dispersive permittivity of the ZnO was fully taken into account and an iterative method was employed to gradually approach the eigenmodes of the cavity. Figure 4b shows the simulated Hz field patterns of the cavity modes at several resonance wavelengths (see insets). Obviously, standing waves are formed in the elongated hexagon microcavity with all of its boundaries serving as totally reflective mirrors. Most importantly, the simulation results fit well with the theoretical prediction of the Cross-WGM model based on the plane wave model. Such optical modes are obviously different from that in the traditional hexagonal WGM microcavity, which should be attributed to the broken hexagonal symmetry of the elongated hexagonal microcavity.

**Figure 5:**
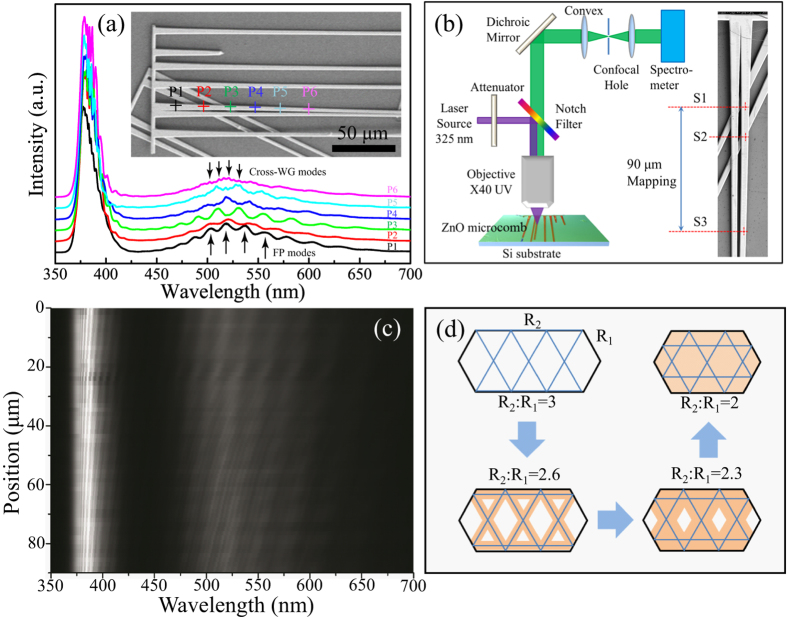
PL spectra of microcavity. (**a**) Room temperature PL spectra collected from the six different locations (P1–P6) marked on the longitudinal axis of the microwire branch by different colour cross in the inset. (**b**) Schematic setup for the PL experiments and the scanning range marked in the inset. (**c**) Spatially resolved PL mapping along c-axis of the branch in (**b**) with TE polarized detection. (**d**) The evolution of the Cross-WGM mode with different ratio of *R*_2_ and *R*_1_.

As mentioned above, the as present ZnO microcombs have a continuously size distribution along the c-axis of the branches, which may provide an ideal test-bed for study their size-dependent optical resonant modes. Figure 5a shows the obtained PL signals collected from the spots marked by the cross in the range of 350–700 nm. It can be seen that the spectra from P1 to P3 were dominant by FP resonant modes. When detect point move to P4, Cross-WGM modes emerged from the FP modes background in visible region, and getting more and more clearly at P5 and P6. The results indicate that there is a transition from pure FP mode to a FP and Cross-WGM modes hybrid emission. To further understand such interesting optical resonant modes, we scanned the excitation beam along the c-axis of the branch (range in 90 μm marked in Fig. 5b, scanning step of 1.5 μm), while detecting the TE polarized emission. From the results shown in Fig. 5c, we see that the UV and visible luminescence band are all clearly modulated as the excitation laser is scanned along the microwire c-axis. The spectra maxima are blueshifed as the sizes of the cavity decreases. And, the transition from FP mode to Cross-WGM is very clearly. According to the SEM, optical images and the optical profilometer data, the R_1_ and R_2_ of the starting point S1, transition point S2 and ending point S3 were identified as follows: R_1_ = 1.54 μm, R_2_ = 4.78 μm, R_2_/R_1_ = 3.10 for S1 point; R_1_ = 1.51 μm, R_2_ = 4.54 μm, R_2_/R_1_ = 3.01 for S2 point; R_1_ = 1.40 μm, R_2_ = 3.32 μm, R_2_/R_1_ = 2.37 for S3 point. It can be find that, the R_2_/R_1_ is gradually decreased from S1 to S3 position. From geometrical analysis, Cross-WGMs form only in the elongated hexagonal cavity involving the shadow marked regions in Fig. 5(d). Only the value of R_2_/R_1_ satisfies the relation of 2 ≤ R_2_/R_1_ ≤ 3, Cross-WGM would be formed inside the ZnO elongated hexagonal cavity. The results are useful for further understanding the physical nature of optical resonant modes of microcavity and developing novel cavity-based optical devices.

## Summary and Conclusions

High quality ZnO microcombs with parallelogram stems and elongated hexagonal branches were fabricated and studied as optical resonators. The formation of such ZnO microcombs was discussed in detail. By using a spatially resolved spectroscopic technique, we directly described the effects of the cross sectional geometry on optical resonant modes experimentally. Cross-WGM and FPM coexist with different polarizations were observed at the branches of microcombs, which is different with the traditional hexagonal WGM microcavity. Such novel optical resonant modes may be due to the broken hexagonal symmetry of the elongated hexagonal microcavity. The observed Cross-WGMs are in perfect agreement with FEM simulations and can be fitted with a plane wave model. Our work would profound understanding of the optical resonant modes in the WGM microcavity with the broken hexagonal symmetry. And, the results are also helpful for us to design high performance micro/nano optoelectronic devices, such as filtering, lasers, sensing and frequency stabilization.

## Additional Information

**How to cite this article**: Dong, H. *et al.* Geometry Dependent Evolution of the Resonant Mode in ZnO Elongated Hexagonal Microcavity. *Sci. Rep.* **6**, 19273; doi: 10.1038/srep19273 (2016).
